# Supplementary material for: Protracted neuronal recruitment in the temporal lobes of young children
Source: Nature. 2023 Dec 20;626(8001):1056–65. doi: 10.1038/s41586-023-06981-x (PMC10901738; doi:10.1038/s41586-023-06981-x)
Supplement: Supplementary file 1 [file 41586_2023_6981_MOESM1_ESM.pdf]

nature portfolio

Corresponding author(s): Marcos Assis Nascimento
Arturo Alvarez-Buylla
Shawn F. Sorrells

Last updated by author(s): Oct 6, 2023

Reporting Summary

Nature Portfolio wishes to improve the reproducibility of the work that we publish. This form provides structure for consistency and transparency in reporting. For further information on Nature Portfolio policies, see our Editorial Policies and the Editorial Policy Checklist.

Statistics

For all statistical analyses, confirm that the following items are present in the figure legend, table legend, main text, or Methods section.

n/a	Confirmed	
☒	☐	The exact sample size (*n*) for each experimental group/condition, given as a discrete number and unit of measurement
☐	☒	A statement on whether measurements were taken from distinct samples or whether the same sample was measured repeatedly
☐	☒	The statistical test(s) used AND whether they are one- or two-sided<br>*Only common tests should be described solely by name; describe more complex techniques in the Methods section.*
☒	☐	A description of all covariates tested
☒	☐	A description of any assumptions or corrections, such as tests of normality and adjustment for multiple comparisons
☐	☒	A full description of the statistical parameters including central tendency (e.g. means) or other basic estimates (e.g. regression coefficient) AND variation (e.g. standard deviation) or associated estimates of uncertainty (e.g. confidence intervals)
☐	☒	For null hypothesis testing, the test statistic (e.g. F, t, r) with confidence intervals, effect sizes, degrees of freedom and P value noted<br>*Give P values as exact values whenever suitable.*
☒	☐	For Bayesian analysis, information on the choice of priors and Markov chain Monte Carlo settings
☒	☐	For hierarchical and complex designs, identification of the appropriate level for tests and full reporting of outcomes
☒	☐	Estimates of effect sizes (e.g. Cohen's *d*, Pearson's *r*), indicating how they were calculated

Our web collection on statistics for biologists contains articles on many of the points above.

Software and code

Policy information about availability of computer code

Data collection	Leica LAS X and Neurolucida v2020.2.4
Data analysis	CellRanger v7, Popscle-freemuxlet (v0.1), Seurat (v4.2), Monocle (v3), DoubletFinder (v2.0.3), DropletUtils (v1.18.1), Neurolucida (v2020.2.4), Imaris (v9.7.1), Graphpad Prism (v9), R (v4.1), ggplot2 (v3.3.5), sf package (v1.0-6), ImageJ (v1.54c), Adobe Photoshop (2023), edgeR(v.3.42)

For manuscripts utilizing custom algorithms or software that are central to the research but not yet described in published literature, software must be made available to editors and reviewers. We strongly encourage code deposition in a community repository (e.g. GitHub). See the Nature Portfolio guidelines for submitting code & software for further information.

Data

Policy information about availability of data

All manuscripts must include a data availability statement. This statement should provide the following information, where applicable:

- Accession codes, unique identifiers, or web links for publicly available datasets
- A description of any restrictions on data availability
- For clinical datasets or third party data, please ensure that the statement adheres to our policy

The raw data for the newly generated sn-RNAseq data, the aligned counts and metadata for each nuclei in the integrated dataset have been deposited in NCBI's Gene Expression Omnibus and are accessible through GEO Series accession number GSE199762. Raw data for additional adult human entorhinal cortex snRNA-seq can be found through GEO Series accession number GSE186538.

Research involving human participants, their data, or biological material

Policy information about studies with human participants or human data. See also policy information about sex, gender (identity/presentation), and sexual orientation and race, ethnicity and racism.

Reporting on sex and gender	The results in this study were based on tissue from donors of both sexes. Detailed information about the sex of individual tissue donors can be found in Supplementary Table 1. 65% (36/55) of donors were male and 35% (19/55) were female. No obvious sex-associated differences were found in our study. A more detailed comparison between sexes is particularly challenging as a much larger number of exact-age-matching donors of both sexes would be needed at multiple ages.
Reporting on race, ethnicity, or other socially relevant groupings	Our study does not group our findings based on race, ethnicity or any other socially relevant categorization.
Population characteristics	Detailed information on sex, age, and clinical history of all samples analyzed can be found in Supplementary Table 1. In short, 27% (15/55) of all samples come from gestational ages, 22% (12/55) from 38 wpc to 42 wpc, 24% (13/55) from 42 wpc to 1 year of age and 27% of samples (15/55) from donors older than 1 year of age.
Recruitment	Participants in this study were research consented postmortem autopsy or epilepsy patients. No active recruitment was implemented. Postmortem human brain samples with no known neuropathological condition were included and were collected from UCSF, UPMC, and the NIH Neurobiobank, all banks in the United States, and the University of Valencia in Spain (see Supplementary Table 1 for details). For epilepsy cases, all tissue was from intractable seizure cases. This is described in the Materials and Methods section, under "Human Tissue Collection" as well as Supplementary Table 1. There is no self-selection bias in these inclusion criteria, however we cannot rule out possible bias from the patient populations and countries sampled or unidentified disease conditions.
Ethics oversight	Human Gamete, Embryo and Stem Cell Research Committee (Institutional Review Board) at UCSF, The Ethical Committee for Biomedical Investigation, Hospital la Fe (2015/0447) and the University of Valencia Ethical Commission for Human Investigation, The Committee for Research on Decedents (CORID) at the University of Pittsburgh. Specimens collected at UPMC had IRB approved research informed consents along with HIPAA authorizations signed by parents or responsible guardians

Note that full information on the approval of the study protocol must also be provided in the manuscript.

Field-specific reporting

Please select the one below that is the best fit for your research. If you are not sure, read the appropriate sections before making your selection.

☒ Life sciences ☐ Behavioural & social sciences ☐ Ecological, evolutionary & environmental sciences

For a reference copy of the document with all sections, see nature.com/documents/nr-reporting-summary-flat.pdf

Life sciences study design

All studies must disclose on these points even when the disclosure is negative.

Sample size	We did not use statistical methods to determine sample size a priori. Our work describes a migration process that was consistently observed at young ages and not in adults across 55 total samples. For the ages and species evaluated this sample size is equivalent to or exceeds sample sizes used in comparable investigations.
Data exclusions	In our single-nuclei RNAseq analyses, barcodes that were assigned as doublets by Freemuxlet our DoubletFinder or nuclei that did not meet our QC parameters (min. UMI count, min. gene count, max. percentage of reads belonging to mitchondrial genes, min. nuclear fraction or nuclei clustered with them)
Replication	Each individual antibody staining was replicated at least 3 times in different experiments across a minimum of 3 sections in each sample. Single-nuclei RNAseq data consisted of two runs: 1) a multiplexed snRNA-seq run where samples of the same cortical region (EC) from 5 different donors (ages ranging 33d-2y) were pooled together and ran in duplicate (in two 2 different wells of a 10x chip), allowing us to confirm that batch effects between wells were minimal. 2) A non-multiplexed snRNA-seq run that did not contain technical replicates for each biological sample, but consisted of additional samples of EC comparable to the ones in the first run and additional samples from LGE, MGE, CGE, and EC stream. These additional samples did not have replicates due the limited availability of samples containing the microdissected regions.
Randomization	Randomization is not relevant since there are no experimental and control groups in this study and all samples were categorized according to their biological age.
Blinding	Samples were not allocated to different groups, so investigators did not have to be blind to group allocations. It is not possible to completely blind investigators to age or region due to obvious cellular differences between regions sampled, the decrease in cell density and large increases in brain size during development. Samples were not quantified in order of age, but were evaluated in a random order.

Reporting for specific materials, systems and methods

We require information from authors about some types of materials, experimental systems and methods used in many studies. Here, indicate whether each material, system or method listed is relevant to your study. If you are not sure if a list item applies to your research, read the appropriate section before selecting a response.

Materials & experimental systems

n/a	Involved in the study
☐	☒ Antibodies
☒	☐ Eukaryotic cell lines
☒	☐ Palaeontology and archaeology
☐	☒ Animals and other organisms
☒	☐ Clinical data
☒	☐ Dual use research of concern
☒	☐ Plants

Methods

n/a	Involved in the study
☒	☐ ChIP-seq
☒	☐ Flow cytometry
☒	☐ MRI-based neuroimaging

Antibodies

Antibodies used

Detailed information on antibodies used, their dilutions, cat. and lot numbers can be found in Supplementary Table 3

Validation

Primary antibodies were selected based on prior validation in human tissue and vendor reported information about antigen target specificity and reactivity in human tissue (See Supplementary Table 3 for manufacturer descriptions about specificity for each antibody). For all antibodies we performed titration experiments (including controls with secondary antibody only) and compared staining patterns to previously published information about developmental or regional expression patterns and intracellular localization of targets.

Animals and other research organisms

Policy information about studies involving animals; ARRIVE guidelines recommended for reporting animal research, and Sex and Gender in Research

Laboratory animals

Rhesus macaque (Macaca mulatta), ages ranging from newborn to 1.5 years of age (Supplementary Table 2)

Wild animals

This study did not involve wild animals

Reporting on sex

Information about sex of animals in this study can be found in Supplementary Table 2. Sex-based analyses were not performed as the main variable in the study was age/developmental stage.

Field-collected samples

This study did not involve samples collected from the field.

Ethics oversight

All experiments were conducted in accordance with Fudan University Shanghai Medical College and University of Pittsburgh guidelines

Note that full information on the approval of the study protocol must also be provided in the manuscript.
